# The Central Role of Amino Acids in Cancer Redox Homeostasis: Vulnerability Points of the Cancer Redox Code

**DOI:** 10.3389/fonc.2017.00319

**Published:** 2017-12-21

**Authors:** Milica Vučetić, Yann Cormerais, Scott K. Parks, Jacques Pouysségur

**Affiliations:** ^1^Medical Biology Department, Centre Scientifique de Monaco (CSM), Monaco, Monaco; ^2^Institute for Research on Cancer and Aging (IRCAN), CNRS, INSERM, Centre A. Lacassagne, Université Côte d’Azur, Nice, France

**Keywords:** cancer, amino acids, redox homeostasis, glutathione, NADPH/NADP^+^

## Abstract

A fine balance in reactive oxygen species (ROS) production and removal is of utmost importance for homeostasis of all cells and especially in highly proliferating cells that encounter increased ROS production due to enhanced metabolism. Consequently, increased production of these highly reactive molecules requires coupling with increased antioxidant defense production within cells. This coupling is observed in cancer cells that allocate significant energy reserves to maintain their intracellular redox balance. Glutathione (GSH), as a first line of defense, represents the most important, non-enzymatic antioxidant component together with the NADPH/NADP^+^ couple, which ensures the maintenance of the pool of reduced GSH. In this review, the central role of amino acids (AAs) in the maintenance of redox homeostasis in cancer, through GSH synthesis (cysteine, glutamate, and glycine), and nicotinamide adenine dinucleotide (phosphate) production (serine, and glutamine/glutamate) are illustrated. Special emphasis is placed on the importance of AA transporters known to be upregulated in cancers (such as system x_c_-light chain and alanine-serine-cysteine transporter 2) in the maintenance of AA homeostasis, and thus indirectly, the redox homeostasis of cancer cells. The role of the ROS varies (often described as a “two-edged sword”) during the processes of carcinogenesis, metastasis, and cancer treatment. Therefore, the context-dependent role of specific AAs in the initiation, progression, and dissemination of cancer, as well as in the redox-dependent sensitivity/resistance of the neoplastic cells to chemotherapy are highlighted.

## Introduction

The potential of targeting redox homeostasis for both cancer prevention and development of novel anticancer treatments has been recognized during past decades. However, despite intensive efforts, development of an effective redox-based therapy remains challenging. A main reason for this is cancer cell plasticity but also our inability to adequately perceive the complexity of redox homeostasis. Namely, antioxidant prophylaxis led to the “antioxidant paradox” (1, 2), while use of chemotherapeutics that compromise the oxidative status of cancer cells encountered resistance (3) and the ability of some cancer cells to upregulate antioxidant protective mechanisms (4). Currently, most attention on targeting redox homeostasis focuses on the attack and downregulation of endogenous antioxidant tumor cell defense mechanisms (5). In this review, we approach cancer redox balance from a different perspective with the main players involving amino acids (AAs).

Although the idea of AA dependency of cancer antioxidant defense (AOD) has received more attention recently, a unified review on this subject is lacking. In 2015, Jones and Sies (6) labeled the nicotinamide adenine dinucleotide (NAD, NADP) and thiol/dysulfide [glutathione (GSH)/glutathione oxidized (GSSG) in the first place] systems together with thiol redox proteome as carriers of the cellular “Redox Code.” According to this principle, spatiotemporal organization of these systems is fundamental for physiology, while its disruption inevitably leads to pathology. Interestingly, accumulating literature indicates that AA availability and metabolism are upstream and superior to these systems, especially in cancer cells. Our review will address this particular aspect of redox regulation in tumors. However, before considering the involvement of AA homeostasis in cancer redox balance, it is necessary to point out some important findings, as well as delusions, that exist in the complex cancer redox field.

## Partially Reduced Oxygen—“Activated” Oxygen

The first steps in understanding oxygen toxicity occurred in the mid-twentieth century when Gerschman et al. (7–9) proposed that the damaging effects of oxygen could be attributed to the formation of oxygen radicals. At approximately the same time, research with [^18^O_2_] and mass spectrometry showed that oxygen atoms from molecular oxygen [O_2_] could be introduced into biomolecules (10, 11). The susceptibility of biomolecules to oxidation gave a biological frame to oxygen toxicity, and together with the discovery of superoxide dismutase [SOD; (12)] fueled research in the field of oxidative damage in biological systems. The term “oxidative stress” was introduced into scientific literature for the first time in 1985 (13).

Now it is clear that the oxidative capacity of molecular oxygen *in vivo* is minimal, but that is not the case for its partially reduced counterparts known as “reactive oxygen species—ROS.” ROS is a term widely used to describe a number of reactive molecules and free radicals derived from molecular oxygen. However, we feel obliged to emphasize the generic nature of this term. ROS includes both radical (superoxide anion radical, $\left[{\text{O}}_{2}^{\cdot -}\right]$; hydroxyl radical, [HO^⋅^]; peroxyl radicals, [ROO^⋅^]; nitric oxide, [NO^⋅^]) and non-radical (hydrogen peroxide, [H_2_O_2_]; hydroxyl anion, [HO^−^]; singlet oxygen, [^1^O_2_]; organic hydroperoxides, [ROOH]) species, which differ significantly in terms of half-life, water/lipid solubility and reactivity. For example, the cellular half-life of lipophobic [HO^⋅^] is only ~10^−9^ s because of its reactivity, compared to ~1 ms for [H_2_O_2_], which also can diffuse through lipid cellular compartments (14). However, use of the common term ROS is sometimes unavoidable (15) due to the complex nature of biological systems, an inability to exactly measure the species generated in a spatiotemporal manner in addition to the so-called theory of “kindling radicals” by which a few primary ROS “inflame” a cascade of ROS amplification by stimulating the sources of secondary ROS (16).

## ROS in Cancer

**Figure 1:**
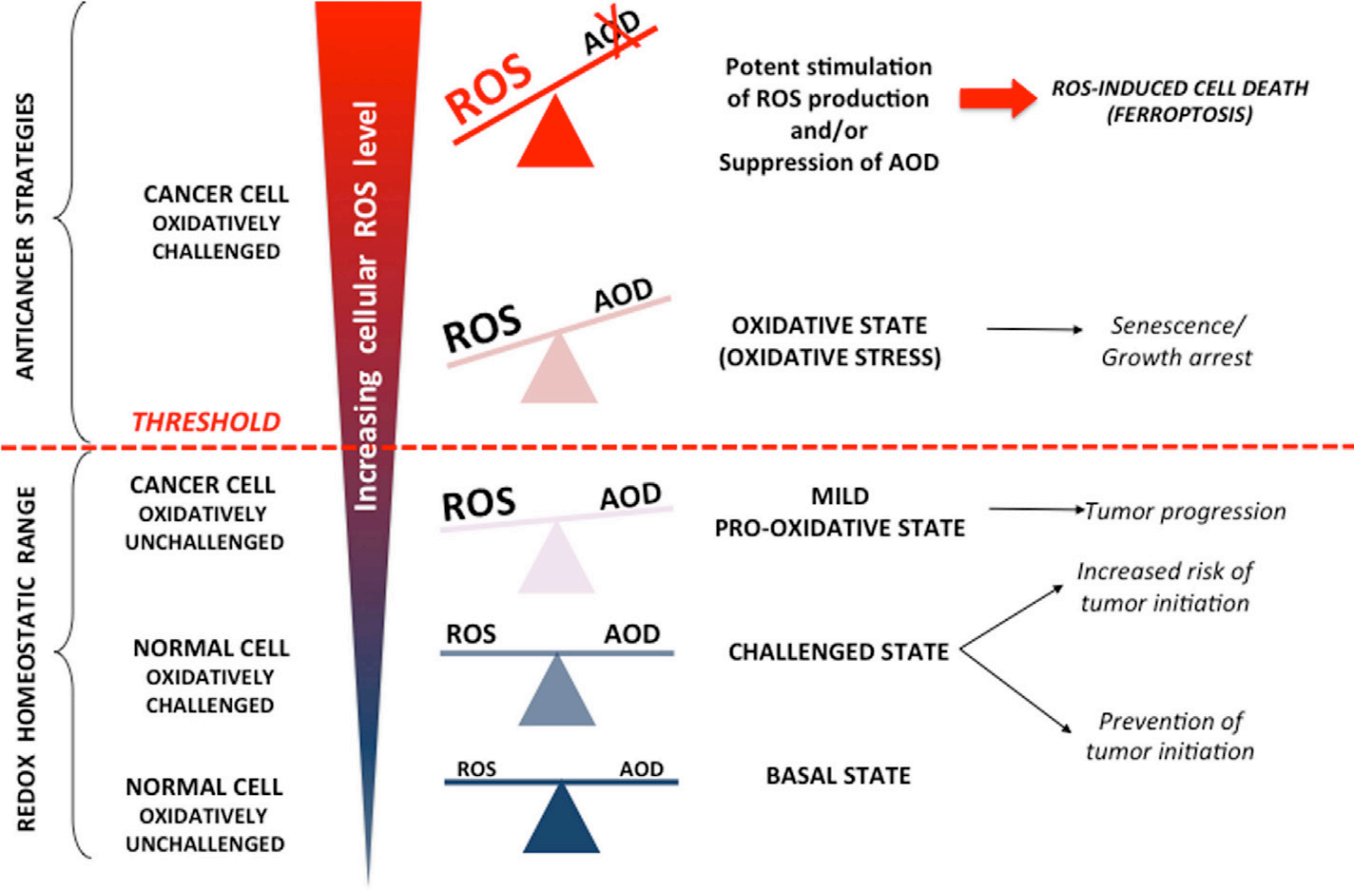
Reactive oxygen species (ROS) can (i) promote cancer, (ii) cause growth arrest, and (iii) be cytotoxic. In normal cells, increased (endogenous or exogenous) oxidative pressure leads to adequate upregulation of cellular antioxidant defense (AOD), which prevent mutagenic events and initiation of cancer formation. However, AOD is not 100% efficient, and thus, these “challenging states” also represent well-known risk factors for cancer development. Once formed, cancer progression seems to be further stimulated by a mild pro-oxidative state due to intensified metabolism, ROS-producing foci, etc. Importantly, this state is still maintained within “redox homeostatic range” thanks to strongly upregulated AOD of cancer cells. However, due to maximized AOD, cancer cells do not support further increase in ROS levels and thus cross the threshold into the state of “oxidative stress.” If ROS level increase further (e.g., due to chemotherapy), the only way for cancer cells to prevent further damage is by decreasing ROS production *via* cell-cycle arrest to repair damage and prevent cell death (cytostatic effects of ROS). However, if ROS burst induces irreversible damage and/or there is not enough components required for repair systems (e.g., glutathione), cancer cells experience programmed cell death or necrosis (cytotoxic effects of ROS).

The terms “ROS” and “cancer” cover a wide range of molecules and diseases, which makes broad generalizations almost impossible. Is it possible, however, to conceptualize some common denominators of the cancer redox state? Widespread opinion is that virtually all malignant cells are in a pro-oxidative state, mostly due to oncogene-driven altered and/or intensified cell metabolism [reviewed in Ref. (17–21)]. However, Halliwell (20) raised important questions regarding ROS measurement in malignant (and other) cells in classical culture conditions that include 21% oxygen and media that is usually deficient in antioxidants/antioxidant precursors and contains free iron ions. These conditions, *per se*, favor ROS generation, and thus special attention should be paid in extrapolating results obtained *in vitro* to the *in vivo* state. Considering this point in combination with current advances in the cancer redox field, a major conclusion that can be drawn is that cancer cells indeed experience mild oxidative pressure in comparison to normal cells (Figure 1) that can help them to exhibit characteristic cancer hallmarks [for detailed review refer to Hornsveld and Dansen (22)].

According to the previous paragraph, it seems that a pro-oxidative state could facilitate initiation and progression of tumorigenesis. However, when reactive and very short living species such as ROS are considered, the situation is not so clear. Accordingly, studies on the effects of antioxidant supplements for cancer prevention and treatment showed opposed and mainly unpromising results, giving rise to confusion and the “antioxidant paradox” (1). Another redox consideration for cancer treatment includes increased ROS levels in cancer cells that already “walk on the edge of oxidative abyss” (23–25). This stand point arises from the very well know concept of hormesis that has been recognized since the XVI century by Paracelsus’s—“*Die Dosis macht das Gift*” or “the dose makes the poison” (26). The concept of hormesis, which revolutionized modern toxicology, claims that the dose–response curve is U-shaped, generally meaning that a drug/stimulus can have opposite effects in small and large doses [for more details refer to Calabrese and Baldwin (27) and papers stemming from it]. Indeed, it has been shown that a mild oxidative state promotes all hallmarks of cancer cells; however, if the threshold is exceeded (reaching the top of the arm of the U-shaped curve), influence of the oxidative environment can easily become anti-carcinogenous, promoting cell-cycle arrest, senescence, programmed cell death, or necrosis (Figure 1). Thus, it has been shown that increased oxidative pressure in the blood, if not adequately balanced by internal AOD, may limit the efficiency of melanoma cells to form distant tumors (28). These results are a textbook example of the antioxidant paradox suggesting how dietary supplementation of antioxidants may promote the metastatic potential of the cancer cells.

The anticancer effects of many conventional therapies, including irradiation and DNA-damaging chemotherapeutics (cisplatin, doxorubicin, gemcitabine, and 5-fluorouracil), rely mostly or partially on increased ROS production, due to mitochondria damage and dysfunction, as well as activation of NADPH oxidase (NOX) enzymes (29–33). However, these treatments encounter resistance with initial response being followed by the development of protective mechanisms against these oxidative/genotoxic insults. The mechanisms of resistance are complex involving drug modification, inhibition, degradation, and/or efflux [for further readings refer to Housman et al. (3)]. In spite of this complexity, the central role that AOD plays in these processes provided the rational for developing anticancer therapies targeting this aspect of cancer redox balance.

## AOD in Cancer

As mentioned previously, oncogenic mutations lead to a pro-oxidative state of cancer cells. However, these cells are still required to maintain ROS levels below the threshold that would become detrimental (Figure 1). Indeed, antioxidant pathways known to respond to increased oxidative pressure in normal cells are constitutively activated in some cancers. The best example is the nuclear factor (erythroid-derived-2)-like 2 (NRF2)-signaling pathway [reviewed elsewhere in great detail (34, 35)]. NRF2 is the main transcription factor regulating expression of AOD enzymes. Under normal conditions, NRF2 is constantly ubiquitinated by Kelch-like ECH-associated protein 1 (KEAP1) and degraded by the proteasome. Oxidants/electrophiles inactivate Keap1 and stabilize NRF2, which then translocates into the nucleus, binds to the antioxidant response element, and activates the transcription of many cytoprotective genes that encode detoxifying enzymes and antioxidant proteins. Constitutive activation of NRF2, due to gain-of-function mutations in NRF2 (36), or loss-of-function mutation in its negative regulator KEAP1, was observed in different types of cancers (37–41). In addition, several tumor-suppressor genes act to repress tumor cell proliferation or cause cells to enter permanent cell-cycle arrest in response to ROS overproduction. These include retinoblastoma, p16^INK4A^, JNK, p38, p53, and forkhead box O. Most of these tumor-suppressor proteins sense changes in the cellular oxidative status and respond accordingly by inhibiting the cell cycle, and thus allowing cells time to recover after oxidative stress, and/or to induce expression of AOD enzymes (22).

Antioxidant defense is divided into enzymatic and non-enzymatic parts. Enzymatic AOD includes enzymes such as SODs, catalases, gluthatione peroxidases (GSH-Px), and glutathione *S*-transferases, as well as redox proteins such as thioredoxins (TRXs), peroxiredoxins, and glutaredoxins. Non-enzymatic AOD components are low-molecular weight compounds such as the key AOD tripeptide glutathione (GSH), vitamins (vitamins C and E), β-carotene, and uric acid. Complementary to these AOD components is the reducing equivalent NADPH that maintains catalases in active forms, serves as a cofactor for TRX and glutathione reductase [which converts oxidized glutathione (GSSG) into its reduced state (GSH)], and acts as a reducing agent for regeneration of glutaredoxins.

The concept of the Redox Code proposed recently by Jones and Sies (6) secludes GSH and NADH/NADPH as main determinants of the dynamic nature of redox signaling and control in multidimensional biological systems. This is even more pronounced in cancer cells due to increased and imbalanced metabolism, mutation accumulation during tumor progression and activated ROS-producing foci (such as defected mitochondria or NOX enzymes). The main reason why GSH and nicotinamide adenine dinucleotide (phosphate) are in the spotlight is the fact that these are the ultimate reducing factors of the cell.

### Glutathione

Glutathione, a tripeptide γ-glutamyl-cysteinyl-serine, appears in two forms: the predominant reduced form (GSH), which reaches millimolar concentrations in the cell, and the minor oxidized form (GSSG), which is estimated to be less than 1% of the total GSH (42). The bulk of GSH is found in the cytosol (~90%), while the rest is localized mainly in mitochondria and the endoplasmic reticulum (ER) (43). GSH functions to detoxify electrophilic compounds including xenobiotics, which makes it central to cellular anticancer drug resistance (44). Owing to the sulfhydryl (−SH) group of cysteine, GSH can serve as an electron donor for reduction of peroxides (reactions catalyzed by GSH-Px) or disulfides. GSH can also directly react with various oxidants in a non-enzymatic manner, although these reaction kinetics are generally very slow (45). In addition, GSH is important in its cysteine-storage function (γ-glutamyl cycle).

Similar to ROS, GSH effects can be pro- or antitumorigenic (46). Although it is important in carcinogen detoxification, increased GSH levels and GSH-dependent biotransformation in many tumors may increase resistance to chemotherapy and radiotherapy (47–50). In addition, high GSH levels are associated with cancer hallmarks such as genomic instability, suppression of apoptosis, invasion, and metastatic activity [for further reading refer to Balendiran et al. (46)].

### NADPH/NADP^+^ Couple

Antioxidant defense is completely ineffective without the NADPH/NADP^+^ cofactor, which serves as a main electron donor for both antioxidant enzymes and catabolic reactions. NADPH supplies reducing equivalents to maintain vital AOD components including the maintenance of active catalase and the regeneration of glutathione, TRX, and glutaredoxin. The NADH/NAD^+^ system is also involved in reversible 2-electron transfer catalysis and is connected with the NADPH/NADP^+^ system by activity of mitochondrial energy-linked transhydrogenase (NNT) (51). However, these two nicotinamide nucleotide systems have somewhat different roles in metabolism. Namely, while NADH/NAD^+^ is involved in catabolism and energy supply, NADPH/NADP^+^ is central for anabolism, defense, and redox homeostasis [reviewed in Ref. (6)]. The redox potential of these two systems also differs significantly in cells. Namely, the cytosolic redox potential of NADH/NAD^+^ is more oxidized (−241 mV) (52, 53) while in mitochondria, it operates at a more negative redox potential (−318 mV) (54), providing reductive force for ATP synthesis. Meanwhile, NADPH/NADP^+^ operates at more negative redox potential than the NAD system both in cytosol (−393 mV) and mitochondria (−415 mV) (53).

The energy-linked mitochondrial enzyme NNT that transfers electrons from NADH to NADPH thus connecting the two systems is of utmost importance in cancers containing mutations in the tricarboxylic acid (TCA) cycle (fumarate hydratase or succinate dehydrogenase) or the electron transport chain (ETC, complex I or III), which have been shown to promote utilization of glutamine by reductive carboxylation (55, 56). Namely, adequate citrate production in these conditions requires high NADPH/NADP^+^ ratios (57), which are achieved by the activity of the NNT (58).

NADPH production occurs *via* the pentose phosphate pathway (PPP), folate metabolism, and malic enzymes (MEs). The importance of AAs for NADPH-producing pathways, especially in cancer cells, is discussed below.

## AAs Sensing from a Redox Perspective

Glucose, AAs, and fatty acids are the crucial building blocks of cellular biomolecules. Tight regulatory mechanisms have evolved to maintain the level of each within homeostatic range. The two main protein kinases involved in sensing and regulation of AA homeostasis are the mechanistic target of rapamycin complex 1 (mTORC1) and general control non-derepressable 2 (GCN2) [for an extensive reviews refer to Bar-Peled and Sabatini (59), Efeyan et al. (60), and Broer and Broer (61)]. Briefly, mTORC1 is a major sensor of specific AAs (Leu, Arg, and Lys), which also receives integrated, growth factors, hormonal, environmental and stress signals regulating growth, and proliferation. Although mechanisms of mTORC1 activation have progressed considerably in the past 20 years, the precise effects of individual AAs on mTORC1 activation have remained elusive. Sabatini’s group has illuminated AA sensing by demonstrating that mTORC1 translocation to lysosomes, is critical for its activation (59). Interestingly, recent studies revealed that this lysosomal localization allows mTORC1 sensing of AA levels (Arg and Gln), not only in cytoplasm but also in lysosomal compartement *via* the lysosomal membrane-resident transport protein SLC38A9 that constitutes a physical and functional part of the AA-sensing machinery (62, 63). Conversely, GCN2-kinase senses AA-uncharged tRNA, resulting in a general suppression of protein translation, paralleled by induction of the mechanisms to increase the cellular AA pool. Data regarding redox dependency of these pathways are still scarce and mechanically unclear.

Earlier studies showed that UV radiation activates mTORC1 signaling through MAP kinase activation by promoting phosphorylation of its downstream target p70^S6k^ in an [H_2_O_2_] concentration and time-dependent manner (64, 65). mTORC1 activation was also observed when cells were treated with oxidizing agents, and surprisingly, even in AA-depleted conditions (66, 67). By contrast, subcellular localization of the mTORC1-interacting protein complex tuberous sclerosis complex at the peroxisome is responsible for mTORC1 repression and autophagy induction in response to ROS (68). Also, the tumor-suppressor ataxia telangiectasia mutated gene, appears to regulate autophagy through repression of mTORC1 in response to oxidative stress (69, 70). Thus, it seems that net effects of ROS on mTORC1 activity are context, time, and dose dependent. However, it should be emphasized that although the AAs leucine, arginine, and lysine are identified as key stimuli for mTORC1 activation, recent work on hepatoma HepG2 cells revealed significant sensitivity of both mTORC1 and GCN2 kinases to cysteine depletion (71). Prompt (within 60 min) inhibition of mTORC1 upon cysteine removal was observed. Considering that the Cys proteome coevolved with advanced [O_2_] sensing and [H_2_O_2_] signaling systems (72–74), this effect of cysteine on mTORC1 from a redox perspective may be of higher importance than the effects of ROS, *per se*.

The main downstream target of activated GCN2 is the eukaryotic initiation factor 2α (eIF2α), whose phosphorylation results in a general reduction of translation initiation, while specific mRNAs containing upstream open-reading frames (e.g., ATF4) are actively translated. However, it has been recognized that GCN2 can be activated by a number of different stresses [osmotic, UV, oxidative (such as [H_2_O_2_]), and ER] independently of AA depletion/imbalance (75–77). Interestingly, although the mechanisms are not yet known, it is recognized that the response of GCN2 to stressors such as [H_2_O_2_] or UV radiation are very fast in comparison to the gradual accumulation of uncharged tRNAs.

In turn, the AA-sensing pathways also influence cellular redox balance. Namely, ATF4, an effector molecule of the GCN2-pathway, also serves as a dimerization partner of the cap “n” collar transcription factor NRF2 (78, 79) promoting resistance to oxidative stress (79, 80). Consistently, it has been shown that mouse fibroblasts lacking *Atf4* depend on supplemental reducing substances, such as glutathione, *N*-acetyl cysteine, or β-mercaptoethanol in their growth media (81). Recent work on HT1080 and A549 tumor cells showed the phosphorylation of eIF2 by protein kinase RNA-like endoplasmic reticulum kinase increases the ability of these cells to cope with increased oxidative pressure in an ATF4-independent manner by activating Akt (82). The importance of the GCN2 kinase in maintaining redox balance was also proved *in vivo*. Mice lacking GCN2 exhibited an increase in protein carbonylation in response to a leucine-imbalanced diet (83).

As for the effect of mTOR on redox homeostasis, a recent study showed that mTORC1 controls ATF4 activity by regulating the translation and stability of its mRNA (84). These results indicate that mTORC1, besides promoting anabolism and consequently increased ROS production, may also contribute to maintenance of the cellular redox equilibrium through “antioxidant properties” of ATF4.

The results listed earlier favor the hypothesis that redox and AA balance are tightly intertwined. How AAs specifically influence the cellular “Redox Code” (GSH and NADPH levels) will be discussed below with special attention placed on the pathways that might represent “vulnerability points” for design of novel anticancer therapeutics.

## Cysteine Levels Determines GSH Levels

**Figure 2:**
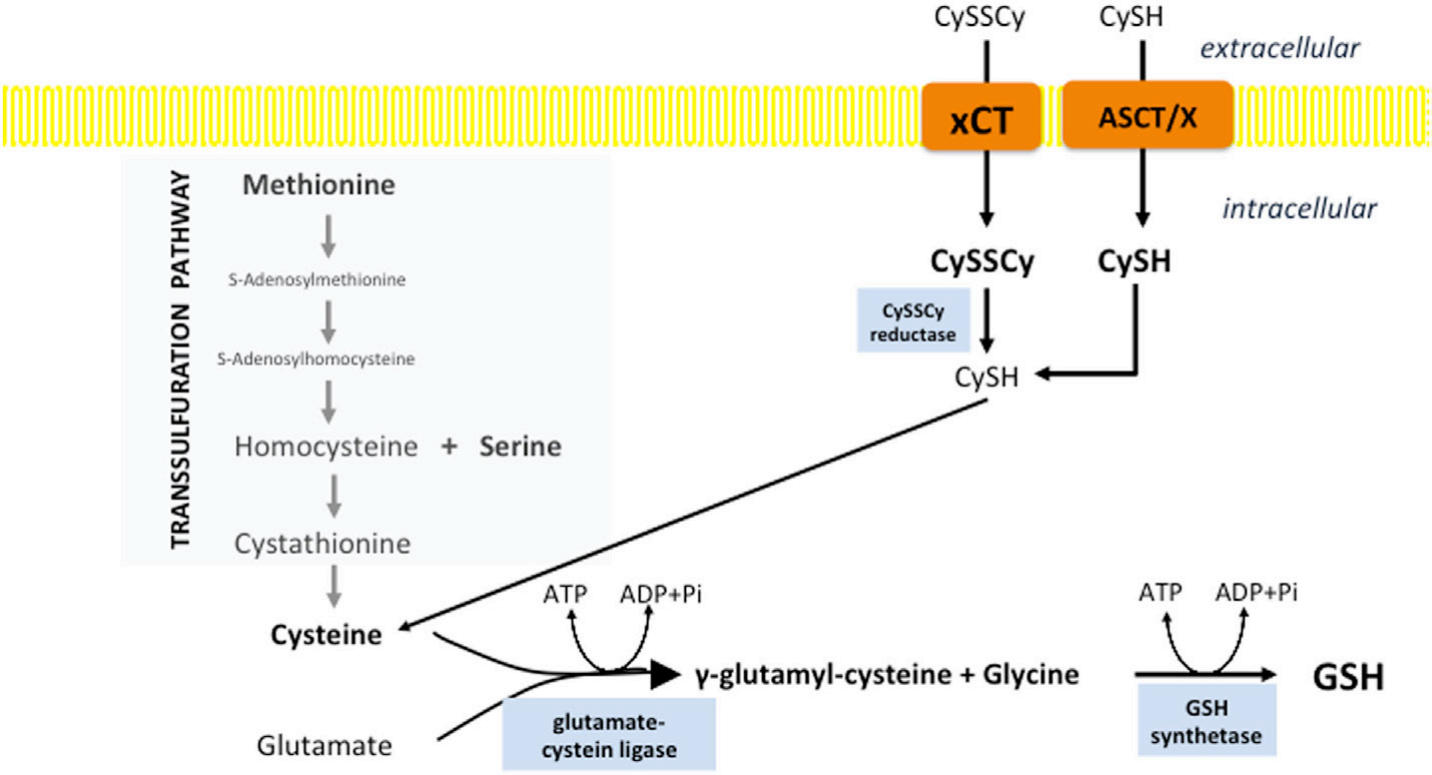
Cystine import is the rate-limiting step in glutathione biosynthesis. Cysteine can be synthesized within the cell through the trans-sulfuration pathway. However, this pathway is often insufficient in cancer cells and therefore cysteine must be imported. Different transporters are involved in the import of the reduced, cysteine (CySH), and oxidized, cystine (CySSCy) form of this semi-essential AA. The heavy-chain transporter subunit of system x_c_-light chain (xCT) seems to play a pivotal role in the import of CySSCy, the predominant form of cysteine in circulation. After import, CySSCy is reduced by cystine reductase and used for different purposes including GSH biosynthesis. Import of cysteine can occur *via* ASCT (alanine/serine/cysteine transporter) and other transporters (x).

Two cytosolic ATP-dependent enzymes are involved in GSH synthesis: glutamate–cysteine ligase (GCL), which catalyzes formation of a particular gamma-peptidic bond between Glu and Cys, and glutathione synthetase. The rate-limiting step in GSH synthesis is the reaction catalyzed by GCL (85). Genetic deletion of the GCL catalytic subunit was lethal in the mouse embryo, while knockout mice for the modifier subunit of the enzyme, although viable and fertile, show a significant decrease of tissue GSH levels (9–16% of wt) (86). The *K*_m_ of mouse GCL for cysteine is estimated at ~0.2 mM (87), which is near the upper limit of typical cellular cysteine concentrations, while the *K*_m_ for glutamate is at or below the cellular glutamate concentration for *Drosophila*, mouse, or human GCLholo enzymes (88–90). Hence, it is not surprising that cysteine is the main regulator of GCL activity, and thus GSH synthesis (Figure 2).

In physiological conditions, cysteine is not an essential AA as it can be synthetized through trans-sulfuration pathways (TSP) from methionine, mainly in the liver. Approximately 50% of the cysteine in hepatic GSH is derived from methionine *via* TSP (91). However, high demand for cysteine in cancer cells, make TSP insufficient (Figure 2). Furthermore, some tumors have shown significantly lower expression of TSP enzymes mostly due to transcriptional silencing (92, 93). Consequently, Cramer and coworkers (94) showed that depletion of cyst(e)ine with pharmacologically optimized cyst(e)inase enzymes induced cell-cycle arrest and cancer cell death due to GSH depletion and ROS accumulation, both *in vitro* and *in vivo*.

### x_c_-Transport System

Multiple tissue-specific transporters are responsible for the import of cystine (CySSCy), the oxidized and predominant form of the AA in circulation (40–50 µM), and/or cysteine, which is present at substantially lower concentrations (8–10 µM) (95–97). However, increasing data in the literature points toward the x_c_-system as being crucial for CySSCy import in cancer cells (Figure 2). The system x_c_- acts as a Na^+^-independent and Cl^−^-dependent antiporter of the anionic forms of cystine and glutamate and is composed of the transporter light-chain (xCT, encoded by *SLC7A11* gene) and a chaperone heavy-chain (CD98hc aka 4F2hc, encoded by *SLC3A2* gene) subunit [for a comprehensive review, see Lewerenz et al. (98)]. Interestingly, although the system x_c_- seems to be a ubiquitous marker of almost all cells cultured *in vitro*, its *in vivo* distribution in humans appears restricted mainly to the CNS, pancreas, fibroblasts, and immune cells (99–105). According to Bannai et al. (106), this induction of the system x_c_- in culture conditions is caused by the high partial pressure of oxygen. Consistent with this hypothesis, prolonged cultivation of fibroblasts in reduced oxygen partial pressure caused a significant decrease in the system x_c_-activity (106).

Considering that AA transporters are necessary for tumor cell proliferation, it is not surprising that xCT is upregulated in many patient samples and tumor cell lines including hepatoma, lymphoma, glioma, colon, breast, prostate, and pancreatic (95, 101, 107–113). Expression of the xCT subunit seems to be under direct control of oncogenes including NRF2 and Ets-1 (114–116). In addition, the promoter region of the *SLC7A11* gene contains an AA response element, which allows the transcription factor ATF4 to enhance expression of xCT in response to AA depletion and/or oxidative stress (115, 117).

System x_c_-light chain mediates import of cystine into cells thus regulating GSH levels (118, 119). Since GSH is the most abundant non-enzymatic antioxidant within the cell, upregulation of xCT satisfies the highly proliferative phenotype of cancer cells. This is supported by complete growth inhibition of lymphoma cells and certain glioma, breast, prostate, lung, and pancreatic cancer cells upon pharmacological inhibition of xCT by sulfasalazine or by the cyclic glutamate analog (109, 111). Besides its role in tumor growth, knockdown or pharmacological inhibition of xCT increased adhesion and inhibited tumor cell invasion *in vitro* and decreased metastases *in vivo* (120). In addition, xCT was shown to associate with CD44v, a major adhesion molecule for the extracellular matrix, which is involved in tumor invasion and metastasis in lethal gastrointestinal tumors (121) along with the metabolic interplay between tumors and host tissue (122). Furthermore, xCT plays a pivotal role in the chemoresistance of tumor cells (123–125), particularly to anticancer drugs that produce high amounts of ROS, such as geldanamycin and celastrol (126, 127).

The importance of the cystine/glutamate antiporter in redox regulation was further implicated in the newly described type of cell death—ferroptosis (128, 129). Ferroptosis is described as an iron-dependent, programmed form of cell death driven by loss of activity of the lipid repair enzyme glutathione peroxidase 4 and subsequent accumulation of membrane lipid peroxides (130). The first described inducer of ferroptosis in Ras-mutated human foreskin fibroblasts was the xCT inhibitor erastin (131). Depletion of intracellular GSH levels due to inhibition of xCT and subsequent increase of ROS levels seems to be sufficient to trigger erastin-dependent cell death. The same results were observed with sulfasalazine, which is another inhibitor of xCT (109, 132). Interestingly, it has been shown that a loss of cysteinyl-tRNA synthetase might prevent erastin-induced cell death by inducing the TSP (133), suggesting that trans-sulfuration can contribute to resistance to inhibition of xCT and ferroptosis induction.

## Serine/Folate Pathway and NADPH Production

Textbooks have stated for years that the main cellular NADPH-producing system is the PPP. Surprisingly, a recent comprehensive study (134) showed that serine-driven one-carbon metabolism (folate cycle) gives almost the same contribution in the NADPH production as the PPP and MEs in proliferating cells. It is also interesting to note that enzymes of both PPP and the serine synthesis pathway (SSP, from which the folate cycle streams out) are induced by NRF2 (135, 136). The function of the folate cycle is ascribed to the collection of one-carbon units from AAs, and subsequent incorporation of these moieties into biomolecules in biosynthetic or methylation reactions. One of the major branching points of the folate cycle is 10-formyl-tetrahydrofolate (10-formyl-THF), which in mitochondria may be used for ATP regeneration [methylene tetrahydrofolate dehydrogenase (MTHFD) reaction], formylation of the mitochondrial initiator *N*-formylmethionine-tRNA or metabolized to [CO_2_], generating NADPH (10-formyl-THF dehydrogenase reaction). On the other side, in cytosol, 10-formyl-THF can be used for purine or NADPH synthesis, while its counterpart 5,10-methylene-THF is used for thymidylate synthesis and homocysteine remethylation in the methionine cycle. In cancer, mitochondrial 10-formyl-THF is mainly used for NADPH production due to overexpression of corresponding enzyme, while in citosol, this reaction is prevented so one-carbon unit, required for purine synthesis, would not be wasted (137, 138). Default mitochondria-to-cytosol directionality of the folate cycle is achived by different expression of enzymes in these compartments, as well as more reductive, i.e., oxidative environment in cytosol and mitochondria respectively (139).

**Figure 3:**
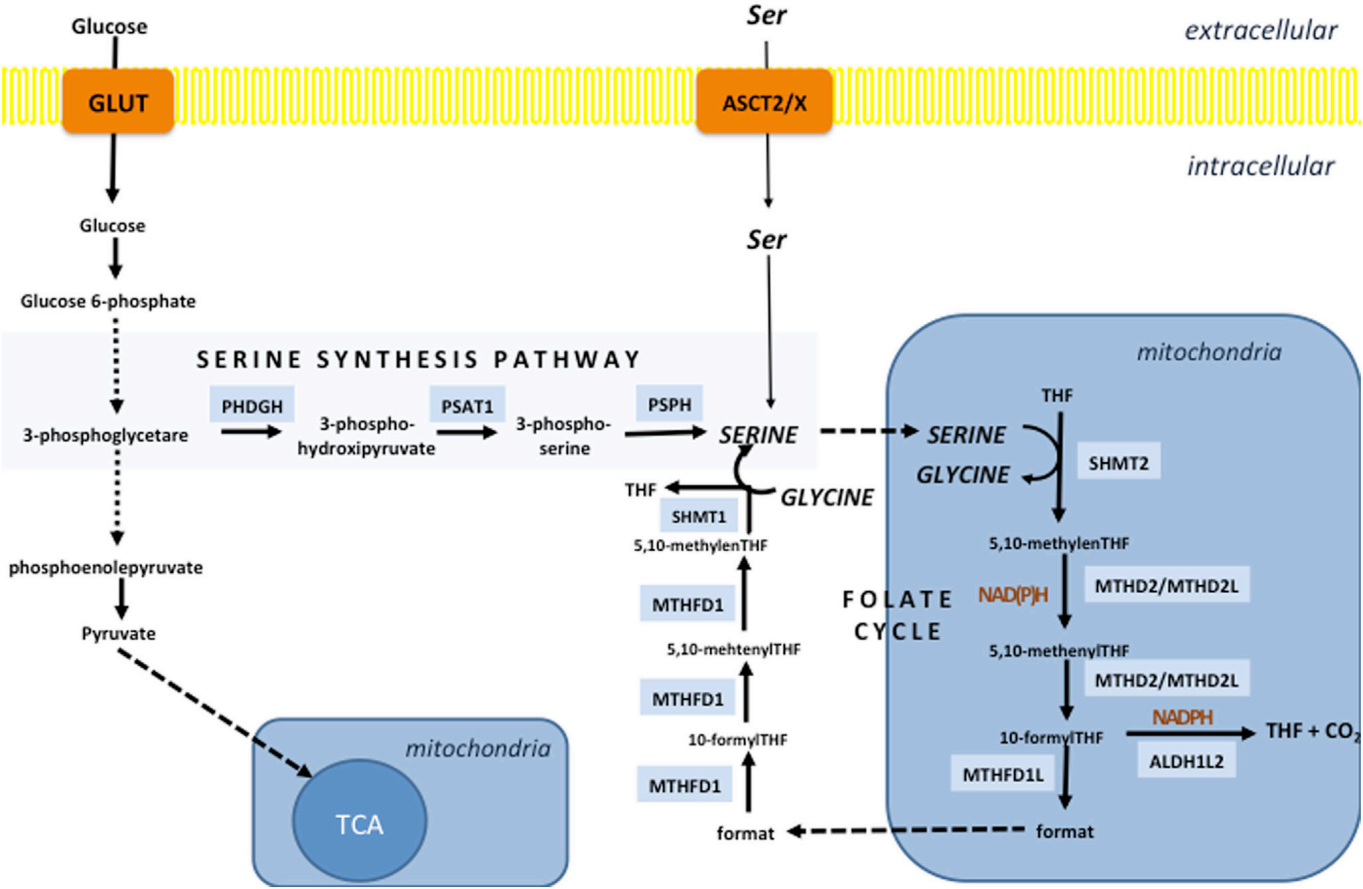
The folate cycle is fueled by the serine synthesis pathway (SSP) and extracellular serine. SSP diverges from glycolysis at the level of 3-phosphoglycerate, which is converted into 3-phospho-hydroxipyruvate by the action of the enzyme phosphoglycerate dehydrogenase (PHDGH) and ultimately to serine following further enzymatic steps. This pathway is of great importance in cancers with mutated or overexpressed PHDGH, while serine import plays a pivotal role in maintenance of the serine cellular balance in cells with unaltered PHDGH activity. The folate cycle in the vast majority of the cells starts in mitochondria by the action of serine hydroxymethyl transferase 2 (SHMT2) which generates glycine and 5,10-methylene-tetrahydrofolate (5,10-methylene-THF). The next reaction can produce NADH or NADPH depending if methenyltetrahydrofolate dehydrogenase 2 (MTHD2) or MTHD2-like (MTHD2L) is used to convert 5,10-methylene-THF into 5,10-methenyl-THF. The same enzyme than generate one-carbon unit—10-formyl-THF, which can be used for ATP production by the enzyme (MTHD1L) or NADPH generation in the reaction catalyzed by 10-formyTHF dehydrogenase (ALDH1L2). If ATP is generated, 10-formylTHF is converted into a format that is transported into the cytosol and used by trifunctional MTHFD1 enzyme to regenerate 10-formylTHF for purine synthesis, 5,10-methylene-THF for thymidylate synthesis and homocysteine remethylation in the methionine cycle. The unidirectionality of the folate cycle seems to be provided by more oxidative mitochondrial redox state that favors use of NAD(P)^+^ by mitochondrial MTHD2(L).

Two mitochondrial reactions of the folate cycle contribute to NADPH production; one is catalyzed by MTHFD, and the other is catalyzed by 10-formyl-THF dehydrogenase (ALDH1L2) (Figure 3). Fan et al. showed that depletion of either of these enzymes decreased NADPH/NADP^+^ and consequently GSH/GSSG ratios and impaired cellular resistance to imposed oxidative stress (134). Similarly, Piskounova et al. showed that redox balancing effects of these enzymes is fundamental for metastatic potential of melanoma cells *in vivo* (28). Namely, this study showed that knockdown of either MTHFD or ALDH1L2 prevents distant metastasis of melanoma cells that encounter high-oxidative pressure in the blood and visceral organs. Besides, it was reported that the first mitochondrial enzyme of the folate cycle, termed serine hydroxymethyl transferase 2 (SHMT2) is essential for maintaining mitochondrial NADPH and GSH level during hypoxia in neuroblastoma cell lines. This study detected a correlation between high expression of SHMT2 and poor prognosis in neuroblastoma patients (140). Expression of SHMT2 in neuroblastoma cells seems to be controlled by the collaborative action of c-Myc and HIF1α. However, numerous oncogenes are reported to affect enzymes of the folate cycle. For example, it is shown that common KRAS mutation associates with increased expression of MTHFD2 in non-small cell lung cancer cell lines (141), while mTORC1-dependent induction of MTHFD2 is reported in both normal and cancer cells (142).

**Figure 4:**
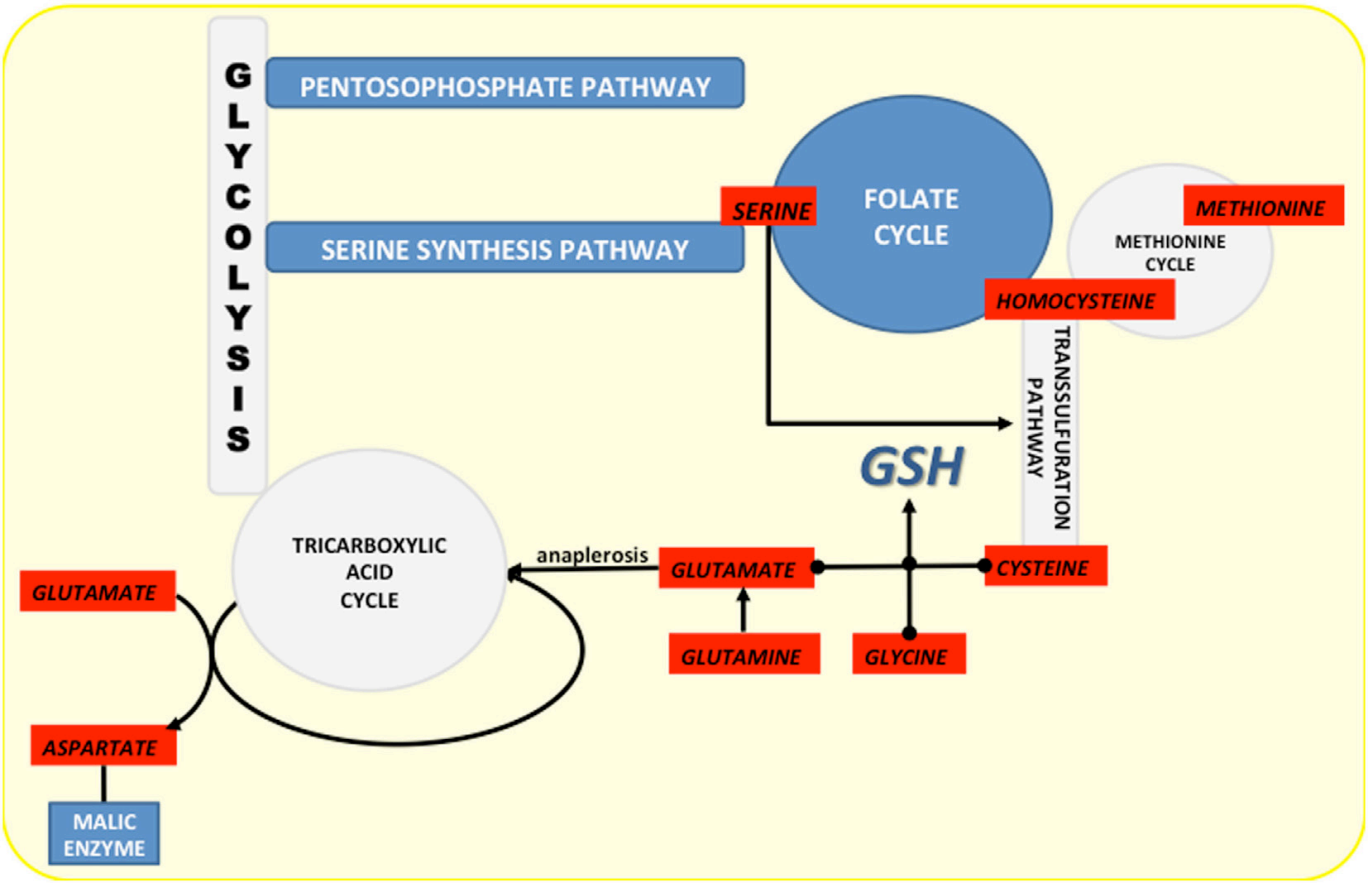
Crossroads of NADPH-producing pathways (marked dark blue) and the pathways from which they diverge or to which they converge (marked light blue). Amino acids involved in these pathways are marked in red.

Besides production of NADPH, the folate cycle contributes to production of GSH by intersecting with the methionine cycle (Figure 4). Considering the role of methionine and homocysteine in the TSP (cysteine synthesis), as well as that glycine is product of serine metabolism (folate cycle), it is not surprising that serine depletion results in reduced level of glutathione (143), while activation of serine synthesis is now well identified as a bypass of glycolysis flux contributing to GSH synthesis (136, 144).

Serine, just like cysteine, can be transported into the cell by different transporters [such as the sodium-dependent transport system ASC that will be mentioned later in the text, and transporter system A, as well as sodium-independent system asc (145, 146)], or synthesized *de novo* from glycolytic intermediate 3-phosphoglycerate through the SSP. Highly proliferating cancer cells both in culture conditions and *in vivo* consume significant amount of exogenous serine (143, 147). Consequently, serine depletion both *in vitro* and *in vivo* decreases proliferation and induces metabolic remodeling, commencing with SSP induction, to replenish cellular serine pool (143).

### Serine Synthesis Pathway

The importance of serine for cancer physiology came from earlier studies that showed increased flux through the SSP in cancer cells (148). However, this was somewhere neglected until the recent discovery that the first enzyme of SSP, phosphoglycerate dehydrogenase (PHGDH), is genetically amplified in breast cancer and melanoma (149, 150), and overexpression of the SSP components are correlated with poorer prognosis in breast cancer patients (151). Consistently, suppression of PHGDH in cell lines characterized with elevated expression of this enzyme decreases cell proliferation and serine synthesis. What is even more interesting is that in non-tumorigenic breast cancer cells, overexpression of PHGDH alone lead to disruption of the acinar cellular morphology and predisposed them to neoplastic transformation (149, 152), making the PHGDH a *bona fide* oncogene (153).

Amplification of PHGDH de-sensitizes tumors to exogenous serine levels but also represents a vulnerability point for potential cancer treatment. Namely, PHGDH knockdown strongly decreased proliferation and some of the SSP outputs [such as α-ketoglutarate (α-KG)] only in cells with amplified PHGDH expression (150). Interestingly, PHGDH also prevents conversion of glycine to serine suggesting that the folate cycle relies exclusively on serine synthesis in PHGDH overexpressing tumors (154). This was demonstrated by PHGDH knockdown decreasing cell proliferation even when exogenous serine was present (154).

Several other oncogenes also induce expression of the SSP enzymes, such as c-Myc and HER2 (155, 156). Also, in line with its involvement in maintaining redox balance, the SSP enzyme expression is induced by NRF2 in an ATF4-dependent manner in NSCLC cells (136). Interestingly, Maddocks and coworkers (143) showed that serine can be a vulnerable point of cancer metabolism even in tumors that do not have multiplication of the *PHGDH* gene, but lack p53. Namely, they showed that the p53–p21 axis is fundamental for metabolic adaptation upon serine deprivation, while loss of p53 in the conditions of serine depletion leads to impaired glycolysis and elevated ROS levels.

Interestingly, pharmacological inhibition of the SSP could also influence flux through the PPP. Namely, inhibition of the SSP would increase intracellular levels of 3-phosphoglycerate, which has been shown to inhibit 6-phosphogluconate dehydrogenase that catalyzes the second step in the oxidative PPP (157).

## Glutamate and NADPH Production

In addition to the PPP and folate cycle, MEs are known to regulate NADPH/NADP^+^ balance, which is seemingly dependent of glutamine metabolism in cancer. One of the main metabolic characteristics of many cancers, besides the Warburg effect (158, 159), is increased consumption of glutamine to the extent where exogenous level of this AA limit tumor cell survival. This “glutamine addiction” has been recognized for more than 50 years (160, 161); however, diverse contributions of glutamine to intermediary metabolism, cell signaling, and gene expression are still not fully understood (162).

**Figure 5:**
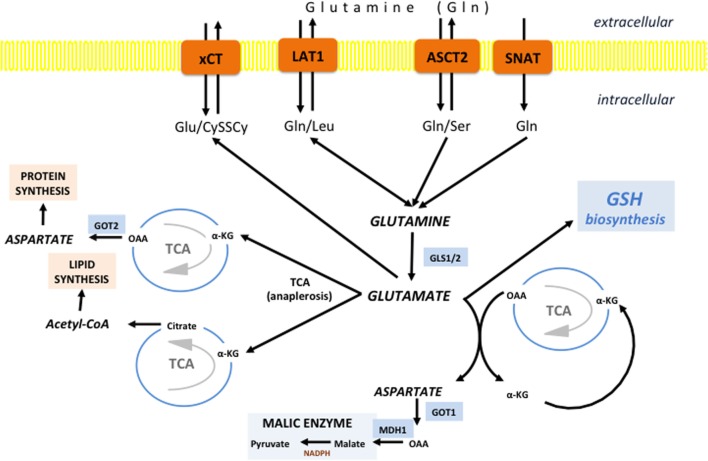
Glutamine/glutamate fates in cancer cells. Different transporters are proposed to fuel the “Glutamine addiction” of cancer cells including alanine-serine-cysteine transporter 2 (ASCT2), SNAT1/2, and L-type amino acid transporter 1 (LAT1). Once inside the cell, Gln can be use for uptake of essential AAs by LAT1. However, the vast majority of Gln is promtly deaminated to glutamate by the action of cytoplasmic or mitochondrial glutaminase (GLS1 and GLS2, respectively). If deaminated in cytosol, Glu is transferred into mitochondria, and there it is further converted into α-ketoglutarate (α-KG) to replenish the tricarboxylic acid (TCA). However, the fate of α-KG can be dual. It can follow normal TCA flow until oxaloacetate (OAA), which is then converted into asparate by aspartate dehydrogenase (GOT2) and translocated into cytoplasm or used for synthesis of asparagine and arginine (protein synthesis). However, if the α-KG is carboxylated to isocitrate and then converted into citrate, citrate is exported into the cytosol where it is used for lipid synthesis in the form of acetyl-CoA. Glutamate-derived aspartate can also be converted into OAA by cytoplasmic GOT1, commonly induced in KRAS-mutated tumors. OAA is then converted first into malate by malate dehydrogenase 1 (MDH1) and then into pyruvate by malic enzyme (ME), generating reducing power in the form of NADPH. Besides involvement in anaplerosis and NADPH production, Glu has an important role as a component of GSH, as well as a substrate for system x_c_-light chain (xCT) in allowing entrance of cystine into the cell.

The vast majority of glutamine in the cell is converted into glutamate either by cytoplasmic glutaminase (GLS1) or by the mitochondrial isoform of this enzyme (GLS2). Glutamate is then converted to α-KG by the enzyme glutamate dehydrogenase. α-KG can then have one of two fates (Figure 5). (1) Canonically, produced α-KG enters the TCA and replenishes it, or (2) it is carboxylated to isocitrate, pushing the TCA in the opposite direction (163).

When glutamine-derived α-KG follows the canonical pathway, the TCA works normally (clockwise) until oxaloacetate (OAA), which is usually converted into aspartate by aspartate transaminase (GOT2) and exported into the cytosol, or alternatively, it can be converted into asparagine and arginine and fuel protein synthesis. Interestingly, a recent study on KRAS-mutated pancreatic ductal adenocarcinoma (PDAC) showed that GOT2 regulates glutamine flux by producing α-KG and aspartate from glutamate and OAA (164). Aspartate is then shuttled into the cytosol where it is converted back into OAA by cytoplasmic GOT1. The OAA produced is converted first to malate and then to pyruvate and NADPH by the action of cytoplasmic malic enzyme 1 (ME1). Considering that KRAS-mutated PDACs have decreased flux through the PPP (165), glutamine-fueled ME1 in these cells may be seen as a major contributor to the NADPH homeostasis. Indeed, ME1 suppression increased ROS accumulation and decreased tumor cell growth both *in vitro* and *in vivo*, while suppressing glutamine utilization and sensitizing cells to oxidative damage (164). Conversely, it remains to be determined if inhibitors of glutamine import or its conversion to glutamate would have the same effects on oxidative status and cell growth.

Oppositely to KRAS, p53 has a negative impact on this NADPH-producing pathway. This was demonstrated by a strong upregulation of MEs (ME1/2) in the absence of functional p53 (166), which were crucial for maintenance of adequate NADPH levels. Here is important to recall the importance of the p53–p21 axis to serine starvation (143) and to anticipate potential resistance mechanisms for serine starvation, in the absence of p53, *via* upregulation of the ME1/2.

### Alanine-Serine-Cysteine Transporter 2 (ASCT2)

Alanine-serine-cysteine transporter 2 (SLC1A5) is a Na^+^-dependent transporter carrying small neutral AAs such as alanine, serine, cysteine, glutamine, and asparagine (*K*_m_ ~20 μM) in addition to long-chain AAs such as threonine, valine, and methionine with lower affinity (*K*_m_ ~300–500 μM). ASCT2 is proposed to play a central role in sustaining cancer cell glutamine homeostasis based on work from Myc-driven cancers, which are particularly addicted to glutamine, and fuel their “glutamine addiction” by promoting high ASCT2 expression (167–169). Also, ASCT2 together with xCT and L-type amino acid transporter 1 (LAT1), comprise the “minimal set” of transporters required for cancer AA homeostasis and the group known to be highly upregulated in cancer (170, 171). Consequently the glutamine import activity of ASCT2 has been proposed to be fundamental for the activity of other AA transporters upregulated in cancer, such as xCT and LAT1 (leucine-for-glutamine exchanger) (171–173). However, recent findings demonstrated that ASCT2 inhibition can be overcame in certain cancer cell types partly by expressing the Na^+^-dependent glutamine transporters system A amino acid transporter 1–2, questioning the functional redundancy for certain AA transporters in tumor growth (174). Regardless, glutamine import (*via* ASCT2 or other transporters) is indeed of great importance for normal functioning of LAT1 and xCT. Recent studies showed that cancer cell glutamine addiction might be a direct consequence of xCT activity, which consumes large amounts of glutamate derived from extracellular glutamine thereby restricting nutrient flexibility of the cell (175, 176).

The importance of glutamine in cancer cells often dominates ASCT2 experimental interpretations. However, it is important to remember ASCT2’s ability to transport other AAs such as serine. As mentioned, some cancer cells remain highly dependent on the uptake of exogenous serine (143). Since ASCT2 display a strong affinity for serine, it would be interesting to investigate the role of this transporter in serine metabolism and redox homeostasis in general. Furthermore, the name of ASCT2: alanine–serine–cysteine transporter may be misleading. Namely, ASCT2 is structurally related to the glutamate transporter and neutral AA transporter ASCT1 and when expressed in *Xenopus laevis* oocyte ASCT2 indeed exhibits Na^+^-dependent uptake of AA similar to ASCT1 (177). However, the same study of Utsunomiya-Tate and collaborators revealed that ASCT2 exhibits different tissue distribution, as well as substrate selectivity and functional properties when compared to ASCT1. Thus, for example, glutamate uptake by ASCT1 is electrogenic, while in the case of ASCT2 lowering pH enhances uptake, which suggests electroneutral uptake. Also, it seems that cysteine is not a substrate for ASCT2, but an allosteric inhibitor of its activity. In accordance to this are recent findings that mark cysteine as a potent competitive inhibitor of ASCT2 that binds to the site different from the one for substrate and induces efflux of glutamine both in the case of proteoliposomes and in intact cells (178).

Considering that the “minimal set” of transporters required for cancer AA homeostasis comprises ASCT2, while its activity/specificity is still rather debatable, it is of utmost importance to continue research on the biology of this very intriguing AA transporter.

## Concluding Remarks

For a long time, the mild pro-oxidative redox state of cancer cells has been recognized as a vulnerable point of these highly metabolically active cells. However, in the context of chemotherapy, we are still struggling to find the adequate approach to the vast majority of ROS-producing therapeutics that encounter cellular resistance and frequent disease relapse. During the past decade, an approach involving suppression of the internal AOD of cancer has attracted more attention. Within highly complex and intertwined AOD system, GSH and NADPH play the most universal and important role in determining the characteristic redox cellular profile. Considering that AA import and metabolism seems to be upstream of these AOD systems, we have emphasized here the specific molecules and pathways that show great, but still insufficiently examined, potential for anticancer therapy from a redox standpoint. In conclusion, the transport and internal synthesis pathways for cysteine, serine, glutamine, and to some extent glycine appear to be the most interesting targets for the development of novel redox-based therapeutics. Targeting AA transport systems (xCT, ASCT2, and SNAT) is promising considering that import of these semi-essential AAs are not required in normal cells, while they are absolutely required for cancer cell survival.

## Author Contributions

MV and JP made substantial contributions to conception and design, revised manuscript critically, and gave final approval of the version to be submitted. YC and SP revised manuscript critically and gave final approval of the version to be submitted.

## Conflict of Interest Statement

The authors declare that the research was conducted in the absence of any commercial or financial relationships that could be construed as a potential conflict of interest.

## Abbreviations

AA(s), amino acid(s); ALDH1L2, 10-formyl-THF dehydrogenase; AOD, antioxidant defense; ARE, antioxidant response element; ASCT2, alanine-serine-cysteine transporter 2; ATM, ataxia telangiectasia mutated gene; eIF2α, eukaryotic initiation factor 2α; ER, endoplasmic reticulum; ETC, electron transport chain; FOXO, forkhead box O; GCL, glutamate–cysteine ligase; GCN2, general control non-derepressable 2; GLS1/2, cytoplasmic/mitochondrial glutaminase; GLUD1, glutamate dehydrogenase; GOT1/2, aspartate transaminase 1/2; GR, glutathione reductase; GS, glutathione synthetase; GSH, glutathione; GSH-Px, glutathione peroxidase; GSTs, glutathione *S*-transferases; GSSG, glutathione oxidized; [H_2_O_2_], hydrogen peroxide; [HO^⋅^], peroxyl radical; [HO^−^], hydroxyl anion; KEAP1, Kelch-like ECH-associated protein 1; LAT1, L-type amino acid transporter 1; ME1/2, malic enzyme 1/2; MTHFD, methylene tetrahydrofolate dehydrogenase; mTORC1, mechanistic target of rapamycin complex 1; NAD(P)H, nicotinamide adenine dinucleotide (phosphate); NNT, energy-linked transhydrogenase; [NO^⋅^], nitric oxide; NOX, NADPH oxidase; NRF2, nuclear factor (erythroid-derived-2)-like 2; [^1^O_2_], singlet oxygen; $\left[{\text{O}}_{2}^{\cdot -}\right]$, superoxide anion radical; PDAC, pancreatic ductal adenocarcinoma; PERK, protein kinase RNA-like endoplasmic reticulum kinase; PHGDH, phosphoglycerate dehydrogenase; PPP, pentose phosphate pathway; PRXs, peroxiredoxins; Rb, retinoblastoma; [ROO^⋅^], peroxyl radicals; [ROOH], organic hydroperoxides; ROS, reactive oxygen species; SHMT2, serine hydroxymethyl transferase 2; SNAT1-2, system A amino acid transporter 1–2; SOD, superoxide dismutase; SSP, serine synthesis pathway; TCA, tricarboxylic acid; THF, tetrahydrofolate; TRXs, thioredoxins; TSC, tuberous sclerosis complex; TSP, trans-sulfuration pathways; CySSCy, cystine (oxidized cysteine); xCT, system x_c_-light chain.
